# Protective effects of decay-accelerating factor on blast-induced neurotrauma in rats

**DOI:** 10.1186/2051-5960-1-52

**Published:** 2013-08-16

**Authors:** Yansong Li, Mikulas Chavko, Jessica L Slack, Bin Liu, Richard M McCarron, James D Ross, Jurandir J Dalle Lucca

**Affiliations:** 1 Immunomodulation of Trauma Program, US Army Institute of Surgical Research, 3650 Chambers Pass, BHT2/Building 3610, Fort Sam Houston, TX 78234, USA; 2 Department of Trauma and Resuscitation Medicine, Naval Medical Research Center, 503 Robert Grant Avenue, Silver Spring, MD 20910, USA; 3 Trauma and Clinical Care Research 59th Medical Wing, Science and Technology Office, 59 MDW/ST, Wilford Hall Ambulatory Surgical Center, 2200 Berquist Drive, Suite 1, Joint Base San Antonio-Lackland, TX, USA

**Keywords:** Blast overpressure, Blast-induced neurotrauma, Complement activation, Blood–brain barrier, Tauopathy, Decay-accelerating factor

## Abstract

**Background:**

Blast-induced neurotrauma (BINT) is the signature life threatening injury of current military casualties. Neuroinflammation is a key pathological occurrence of secondary injury contributing to brain damage after blast injury. We have recently demonstrated that blast-triggered complement activation and cytokine release are associated with BINT. Here, we evaluated if administration of the complement inhibitor recombinant human decay-accelerating factor (rhDAF) is beneficial on neuroinflammation and neurodegeneration in a rat model of moderate BINT. Administration of rhDAF after exposure to moderate blast overpressure (BOP, 120 kPa) mitigated brain injury characterized by neuronal degeneration. rhDAF treatment reduced complement hemolytic activity at 3 hours and tissue complement deposition at 3, 24, and 48 hours as well as systemic and local cytokine release at 24 hours post BOP. Furthermore, rhDAF protected blood–brain barrier (BBB) integrity and reduced cytotoxic edema. Interaction between complement cleavage component, C3a and C3a receptor and tau phosphorylation were also attenuated in rhDAF treated animals at 3 and 24 hours after BOP. These novel findings suggest early complement targeted inhibition as a new therapeutic strategy to decrease neuroinflammation and neurodegeneration after blast TBI.

**Result:**

Administration of rhDAF after exposure to moderate blast overpressure (BOP, 120 kPa) mitigated brain injury characterized by neuronal degeneration. rhDAF treatment reduced complement hemolytic activity at 3 hours and tissue complement deposition at 3, 24, and 48 hours as well as systemic and local cytokine release at 24 hours post BOP. Furthermore, rhDAF protected blood–brain barrier (BBB) integrity and reduced cytotoxic edema. Interaction between complement cleavage component, C3a and C3a receptor and tau phosphorylation were also attenuated in rhDAF treated animals at 3 and 24 hours after BOP.

**Conclusion:**

These novel findings suggest early complement targeted inhibition as a new therapeutic strategy to decrease neuroinflammation and neurodegeneration after blast TBI.

## Background

Traumatic brain injury (TBI) is a leading cause of death and disability, contributing to one-third of all injury-related deaths in the United States, and a significant cause of loss of productivity [1]. It is estimated that 10-20% of returning veterans sustain TBI while deployed, making TBI the hallmark injury of current military casualties. In these cases, most TBI results from exposure to blast overpressure (BOP) from explosive devices, resulting in cell death and neuronal dysfunction. Furthermore, military casualties exposed to blast often experience delays in medical evacuation to higher echelons of care, with time intervals ranging from 1 hour to several days [2]. During this time frame, secondary molecular responses promote further neuronal injury and consequently, neurodegeneration. The development of secondary injury subsequent to the primary injury provides a window of opportunity for therapeutic intervention to prevent progressive tissue damage after traumatic brain injury (TBI) [3]. Neuroinflammation is a well-established secondary injury mechanism that largely contributes to damage observed after TBI [4].

Neuroinflammation following TBI is mediated by an early activation of the innate immune system [5]. The complement system, a key player in innate immunity, may participate as an important early mediator of neuroinflammation and neurodegeneration after TBI. Various studies in animal models and clinical studies have demonstrated elevated levels of complement components and complement activation fragments in serum, cerebrospinal fluid (CSF), and brain parenchyma after TBI, with broad parenchymal deposition of complement components [6-9]. In both clinical and experimental studies, severe secondary insults in TBI were observed in parallel to pronounced complement activation, specifically increased levels of the complement activation product, C5b-9 [10]. These secondary injuries include the recruitment of inflammatory cells into the intrathecal compartment, the induction of blood brain barrier (BBB) dysfunction by activated complement C3a and C5a, the induction of neuronal apoptosis through the neuronally expressed C5a receptor (C5aR), and homologous cell lysis mediated by C5b-9 [11]. Prior studies from our lab report that complement activation and C3a-C3aR interaction are associated with hypoxia-induced disruption of neuronal networks, loss of dendritic spines, and neuronal apoptosis [12].

Genetic and pharmacological manipulation of both complement levels and complement activation in mouse models of TBI are reported to be highly neuroprotective, suggesting the complement cascade is a critical target for managing post-TBI tissue damage. Mice deficient in the C3 gene [13,14] or overexpressing complement regulatory proteins such as Crry or vaccinia virus complement control protein, exhibit significant neuroprotection, attenuated BBB disruption, and improved neurological outcome after injury [15,16]. Inhibition or deficiency of other complement components have also been demonstrated to be effective in ameliorating injury, including inhibition of C1 by C1-inhibitor (C1-INH) [17], C4 deficiency or inhibition by C4 antibody [18], C5aR blockade [19], and factor B deficiency [20] or inhibition by factor B antibody [21]. In our most recent work, we found early complement activation was dramatically increased profound after blast injury. In addition, higher systemic and local levels of C5b-9 were detected as early as 3 h and persisting for up to 48 h were associated with BINT in rats exposed to blast overpressure (BOP) [22]. These results strongly suggest early administration of complement inhibitors as a novel and viable pharmaceutical strategy to mitigate BINT.

Administration of decay-accelerating factor (DAF), an inhibitor of alternative and classical complement activation pathways has been shown to protect neurons from hypoxia-induced disruption of neuronal networks, loss of dendritic spines, and neuronal apoptosis in cultured primary neuronal cells [12]. In the study presented here, we hypothesized that the complement system plays a critical role in the development of secondary injury and early administration of DAF would be neuroprotective in a rat model of blast-induced neurotrauma.

## Methods

### Animals

Adult pathogen-free male Sprague–Dawley rats weighing 250 to 300 g (Charles River, Wilmington, MA) were used in this study. Experiments were conducted in compliance with the Animal Welfare Act at an AAALAS accredited institution and in accordance with the principles of the Guide for the Care and Use of Laboratory Animals. The study was approved by the Naval Medical Research Center Institutional Animal Care and Use Committee.

### Reagents

Recombinant human DAF (rhDAF) and biotinylated anti-human DAF was obtained from R&D systems (Minneapolis, MN). Chicken anti-mouse C3/3a, mouse anti-rat endothelial cells, mouse anti-rat aquaporin-4, and rabbit anti-p-tau (phosphor T205) antibodies were obtained from Abcam Inc. (Cambridge, MA). Mouse anti-rat C3a receptor (C3aR) and mouse anti-rat C5b-9 antibodies were acquired from Hycult Biotech Inc (Plymouth Meeting, PA). Biotinylated goat anti-rat IgG was from Vector Laboratories (Burlingame, CA). Streptavidin Alexa Fluor 488, goat anti-mouse Alexa Fluor 488-, goat anti-rabbit 594-, and goat anti-chicken 594-conjugated secondary antibodies, and ProLong Gold antifade reagent were from Invitrogen (Carlsbad, CA). Bio-Plex Pro™ rat cytokine multiplex assay kit was purchased from Bio-Rad laboratories (Hercules, CA).

### Experimental design and administration of DAF

Exposure to blast was conducted as previously described [22]. Briefly, adult male rats were anesthetized with intraperitoneal injection of ketamine/xylazine (60/5 mg/kg) and randomly assigned to each experimental group. Anesthetized animals were placed into the end of the expansion chamber of a compressed air-driven shock tube (2.5 ft compression chamber connected to a 15 ft expansion chamber) and fixed into a holder to restrict any body movement from blast impact and prevent subsequent secondary or tertiary blast injuries. Animals were subjected to a single blast exposure with mean peak overpressure of 120 ±7 kPa with their right side ipsilateral to the direction of the BOP. Animals were randomly assigned to one of seven experimental groups: 1) Control, animals underwent anesthesia, suspension, and time delays except for BOP (n = 8); 2) BOP-3 h, animals were subjected to BOP followed by a bolus injection (vehicle, 0.5 ml of saline) via tail vein 30 min post BOP and recovered for 3 h (n = 8); 3) BOP-24 h, animals were exposed to BOP + vehicle and recovered for 24 h (n = 8); 4) BOP-48 h, animals underwent BOP + vehicle and recovered for 48 h (n = 5); 5) DAF-3 h, animals were exposed to BOP followed by a bolus of rhDAF (50 μg/kg body weight) injection via tail vein 30 min post BOP and recovered for 3 h (n = 6); 6) DAF-24 h, animals were exposed to BOP followed by a bolus of rhDAF (50 μg/kg body weight) injection via tail vein 30 min post BOP and recovered for 24 h (n = 8); and 7) DAF-48 h, animals were exposed to BOP followed by a bolus of rhDAF (50 μg/kg body weight) injection via tail vein 30 min post BOP and recovered for 48 h (n = 5).

### Blood and tissue collection

Animals were euthanized with an overdose of pentobarbital at indicated time points, and blood was withdrawn by cardiac puncture. Serum samples were collected by centrifuging at 4000 rpm for 10 minutes and stored at - 80°C until use for systemic cytokine analysis and CH50 assay. The brains were quickly removed, and cut at 3-mm thickness. The cerebral slices were frozen, and stored at -80°C until use for measuring tissue levels of cytokines. The cerebral slices were fixed in 10% formalin solution for histological evaluation, or fixed in 4% paraformaldehyde for immunohistochemical staining.

### Histological evaluation

Ten percent formalin-fixed tissues were embedded in paraffin. Coronal sections were then cut, and stained with hematoxylin-eosin (H&E). Five random histologic images were recorded at × 400 magnifications under an Olympus AX80 light microscope (Olympus, Center Valley, PA) and graded by a pathologist blinded to the treatment group.

Frontal cortical and hippocampal (dentate gyrus, DG) damage was assessed by five distinct morphological parameters: neuronal morphological changes (shrinkage of the cell body, pyknosis of the nucleus, disappearance of the nucleolus, and loss of Nissl substance, with intense eosinophilia of the cytoplasm), neuronal loss, cytotoxic edema, vasogenic edema, and inflammatory cell infiltration in the brain cortex. The changes were scored according to their extent (score 0, 1, 2, 3, and 4 for an extent of 0%, < 25%, 25–50%, 50–75%, and 75–100%, respectively) and the severity of the injury (score 0 = normal histology, score 1 = slight, 2 = mild, 3 = moderate, and 4 = severe alterations). The injury score represents the sum of the extent and the severity of injury.

### Immunohistochemical staining

After 4% paraformaldhyde fixation, brains were transferred to 20% sucrose (w/v) in PBS overnight at 4°C, followed by freezing in Tissue-Tex OCT mounting medium. Coronal frozen sections were cut at 5-μm thickness with a cryostat and mounted onto glass slides. The tissues where fixed in cold acetone or 4% paraformaldehyde for 20 min and permeabilized with 0.2% Triton X-100 in PBS for 10 min. The sections were blocked with 2% bovine serum albumin and incubated with the primary antibodies overnight at 4°C. After washing, the sections were incubated with the appropriate secondary antibodies labeled with Alexa Fluor 488 or 594 for 1 h at room temperature. After washing, the sections were mounted with ProLong Gold antifade solution containing 4, 6’-diamidino-2-phenylindole and visualized under a Radiance 2100 confocal laser scanning microscope (Bio-Rad,Hercules, CA) at × 200 or × 400 magnification. Negative controls were conducted by substituting the primary antibodies with corresponding immunoglobulin isotypes. Captured digital images were processed by Image J software (NIH, Bethesda, MD).

### Immunofluorescent quantification

This procedure is based on a modified method as described previously [23]. Briefly, four to six images from each animal were calibrated using the Adobe Photoshop software and adjusted until only the fluorescent deposits and no visible tissue background. The image was changed to black-and-white pixels with black representing deposits of the target proteins and white representing nonstained areas of the image using the Image J software. Using the image Adjust Threshold command, the image was then changed to red and white (fluorescent deposits were in red). Image analysis resulted in the red total area in pixels squared. Values for total area for all animals in each group were averaged to give the average area of fluorescent deposit.

### Fluoro-jade B staining

Coronal frozen sections were cut at a thickness of 20 μm with a cryostat. The sections were collected in 0.1 M phosphate buffer (PB) and mounted onto 1% gelatin coated slides and air dried on slide warmer at 50°C for 30 min. The slides were immersed in a solution containing 1% sodium hydroxide in 80% alcohol for 5 min followed by 2 min in 70% alcohol and 2 min in distilled water. The slides were then transferred to a solution of 0.06% KMnO_4_ for 10 min and washed 3 times in ultrapure water for 1 min each. The slides were subsequently stained in 0.001% Fluoro-Jade B (Histo-Chem Inc., Jefferson, AR) in 0.1% acetic acid for 15 min. and rinsed 3 times in ultrapure water for 1 min each. The slides were allowed to air dry on slide warmer at 50°C for 10 min and cleared by immersion in xylene for 1 min before coverslipping with DPX (Sigma, St. Louis, MO). Digital images were collected on a fluorescent microscope at × 200 magnification.

### Tissue protein extraction

Frozen brain tissue samples were thawed, washed with ice-cold phosphate-buffered saline (PBS), suspended in radio-immunoprecipitation assay (RIPA) buffer containing protease inhibitors (2 μg/ml of aprotinin, 10 μM of leupeptin, 1 mM of phenylmethylsulfonyl fluoride), and minced on ice. Brain tissue was homogenized on ice and clarified at 13,000 rpm for 15 min at 4°C. Aliquots of supernatant were used to determine protein concentration by Bio-Rad DC protein assay kit and cytokines (Bio-Rad Laboratories, Hercules, CA).

### Cytokine quantification

Cytokine levels in the serum and brain tissue were measured by Bio-Plex Pro™ rat cytokine multiplex assay kit according to the manufacturer’s instructions using the Luminex^®^ 200™ system (Invitrogen, Carlsbad, CA).

### CH50 assay

Serum complement activity was determined based on hemolytic activity. Briefly, antibody-sensitized *Gallus gallus domesticus* red blood cells (Colorado Serum Company, Denver, CO) were incubated for 1 h at 37°C with serial dilutions of serum samples in gelatin-Veronal buffer (pH 7.3). After centrifugation, absorbance of the supernatant was determined at 405 nm, and the serum concentration inducing 50% of complement hemolytic activity was determined as CH50 value.

### Statistical analysis

Data are expressed as mean ± standard error of the mean (SEM). One-way analysis of variance (ANOVA) followed by Bonferroni or unpaired *t*-test was performed using GraphPad Prism^®^ (5.0, GraphPad Software, San Diego, CA). P value <0.05 was considered as significant.

## Results

### Recombinant human DAF deposits in rat cortex and hippocampus

**Figure 1:**
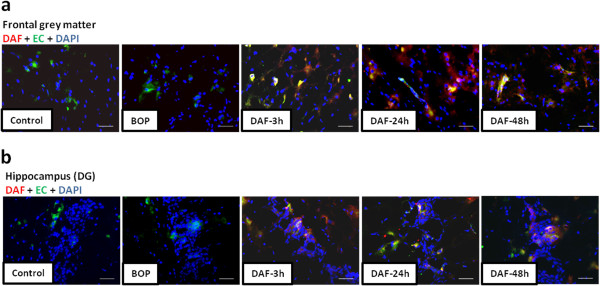
**Deposition of rhDAF in the brain cortex and hippocampus of animals treated with rhDAF.** Representative photomicrographs of frontal grey matter **(a)** and hippocampal DG **(b)** from the frozen sections of rat brains immunostained by anti-human DAF (red) and anti-endothelial cell (EC, green) antibodies. Original magnification of × 200. Scale bar, 200 μm. n = 8 for control, 3 and 24 h experimental groups. n = 5 for 48 h experimental groups.

Deposition of rhDAF in the brain was determined by immunohistochemical staining using anti-human DAF antibody. As shown in Figure 1a and b, rhDAF deposition was observed in the cortex and hippocampus of DAF-treated animals. Deposition of rhDAF appeared to be associated with the cerebral endothelium. No rhDAF deposition was evident in the controls and non treated animals.

### Administration of rhDAF mitigates brain neuronal degeneration in rats subjected to BOP

**Figure 2:**
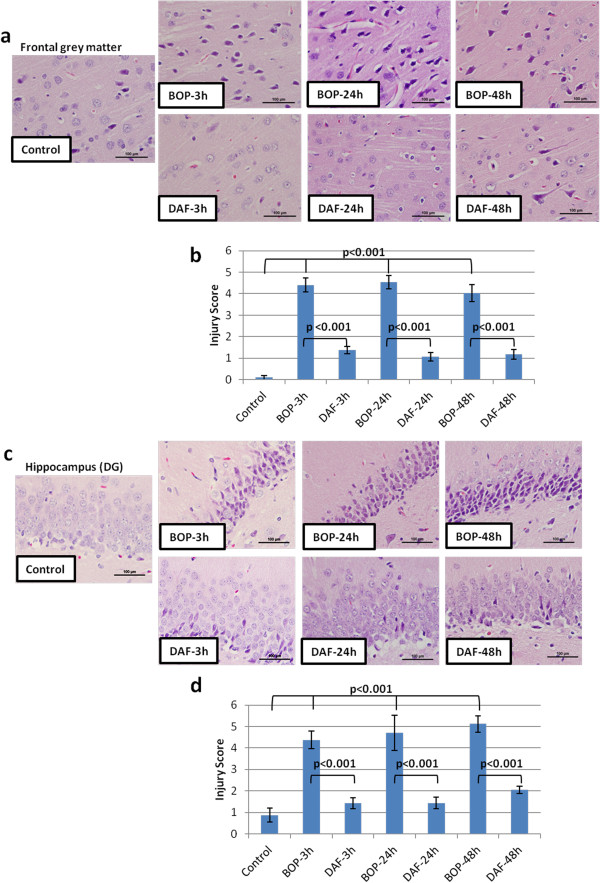
**Early treatment of DAF attenuates brain injury of rats exposed to 120 kPa BOP. a** and **c**: Representative photomicrographs of coronal paraffin sections stained with H&E of control, BOP-3 h, BOP-24 h and BOP-48 h untreated and treated with rhDAF (50 μg/kg) 30 min post blast exposure. **a**: brain frontal cortical grey matter and **c**: hippocampal dentate gyrus, DG. Original magnification of × 400. Scale bar, 100 μm. **b** and **d**: Mean brain injury scores, n = 8 for control, 3 and 24 h experimental groups, n = 5 for 48 h experimental groups. Injury scores calculated using the criteria as described in the Methods. Graphs expressed as mean ± SEM, and compared using one-way ANOVA followed by Bonferroni test.

Histological analysis of the frontal cortex in the ipsilateral and contralateral sides after a recovery period of 3, 24 and 48 h following blast exposure revealed microscopic changes in the grey matter (Figure 2a and b). BOP exposure resulted in bilateral capillary damage, brain edema and neural morphological changes characterized by cell body shrinkage and nuclear pyknosis. These changes were significantly attenuated by an early bolus administration of rhDAF (50 μg/kg), 30 minutes after blast exposure. The beneficial effects of DAF administration were seen throughout the recovery periods of 3, 24 and 48 h after the blast exposure.

As shown in Figure 2c and d, histopathological analysis of the ipsilateral and contralateral dentate gyrus demonstrated significant changes in neuronal morphology in comparison to controls at 3, 24 and 48 h post BOP. These changes were characterized by bilateral neuronal loss and pyramidal cell alteration with morphologic features consisting of shrinkage of cell body, pyknois of nucleus, disappearance of nucleolus, and loss of Nissl substance. Notably, the morphological alteration of the hippocampus from BOP was markedly improved in animals treated with rhDAF (Figure 2).

**Figure 3:**
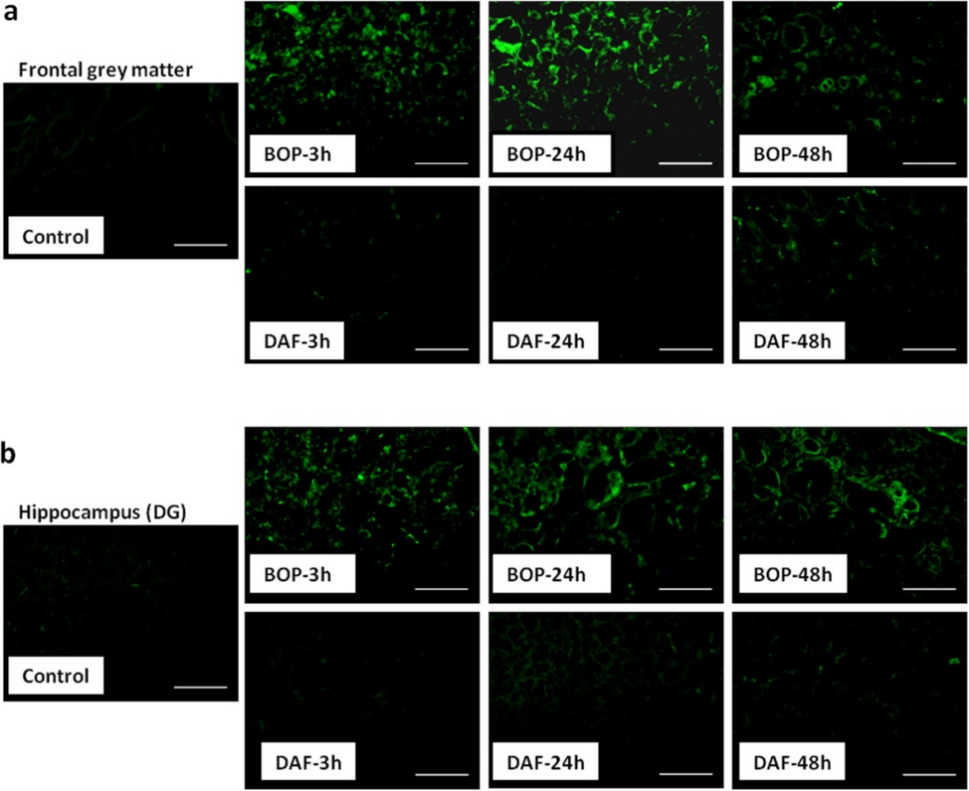
**Early administration of DAF improves BOP-induced neurodegeneration.** Coronal frozen sections of 20-μm thickness were stained with Fluoro-Jade B as described in the Methods. Representative Fluoro-Jade B staining in frontal grey matter **(a)** and hippocampus (DG) **(b)** following BOP. n = 8 for control and experimental groups. Original magnification of × 200. Scale bar, 200 μm.

Similar to what was observed with H&E staining, a significant increase in Fluoro-Jade B staining in both the cortex and hippocampus from 3 h to 48 h after BOP, peaking at 24 h was observed (Figure 3a and b). In contrast, rhDAF treatment significantly reduced Fluoro-Jade B positive cells in the cortex and hippocampus after BOP (Figure 3a and b). Fluoro-Jade B is a specific marker for the histological staining of neurons undergoing degeneration. Thus, these results indicate that the BOP-induced morphological alterations seen in the hippocampus and cortex are primarily due to neurodegeneration.

### rhDAF attenuates complement activation and deposition in rats exposed to BOP

**Figure 4:**
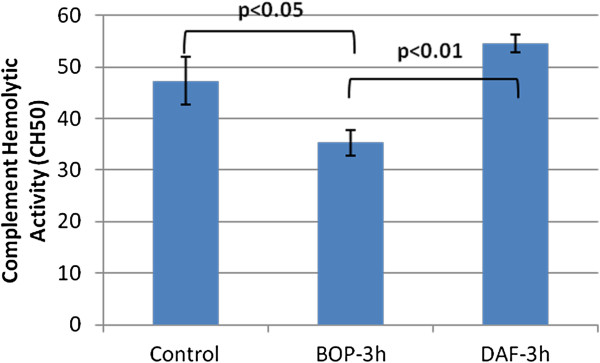
**Effect of DAF on complement function after blast exposure.** Complement function was measured by hemolytic activity (CH50) assay. Group data is expressed as mean ± SEM and compared using one-way ANOVA followed by Bonferroni test. n = 8.

BOP-induced changes in complement function were assessed by complement hemolytic activity assay. As shown in Figure 4, complement hemolytic activity was significantly reduced in the serum of blast exposed animals obtained at 3 h, indicating that complement activation occurred after BOP exposure. Complement hemolytic activity was virtually abolished 24 h after BOP and returned to pre-BOP exposure levels after 48 h post BOP (data not shown). Complement hemolytic activity significantly increased in rats treated with rhDAF at 3 h after BOP demonstrating complement inhibition by rhDAF.

**Figure 5:**
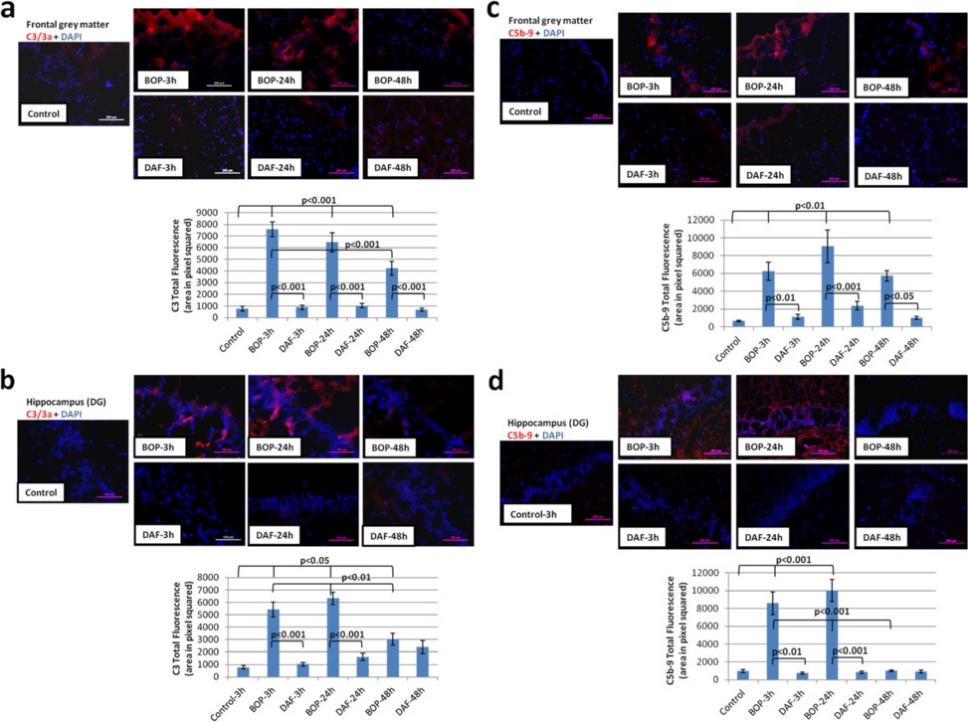
**Early administration of DAF decreases C3 and C5b-9 deposition in brain tissue from BOP exposed animals.** Representative photomicrographs of brain frozen sections were stained with anti-C3/3a and C5b-9 antibodies (top), and the total fluorescence of C3 and C5b-9 deposition (bottom). Frontal grey matter **(a and c)** and hippocampus (DG) **(b and d)**. Original magnification of × 200. Scale bar, 200 μm. Group data is expressed as mean ± SEM and compared using one-way ANOVA followed by Bonferroni test. n = 8 for control, 3 and 24 h experimental groups. n = 5 for 48 h experimental groups.

Previously we have demonstrated higher levels of complement activation and deposition in rat brains at 3 and 48 h after exposure to 120 kPa BOP [22]. As expected, increased deposition of C3 and C5b-9 at the superficial layers of the cortex as early as 3 h and lasting 24 and 48 h after blast was observed (Figure 5a and b). Deposition of C5b-9 was seen after 3 and 24 h post blast in the hippocampus, whereas limited deposition was found at 48 h after blast. The deposition of complement components in the cortex and hippocampus after blast exposure was significantly attenuated with the administration of rhDAF (Figure 5).

### DAF decreases expression and interaction of C3a and C3aR in rat brain after BOP

**Figure 6:**
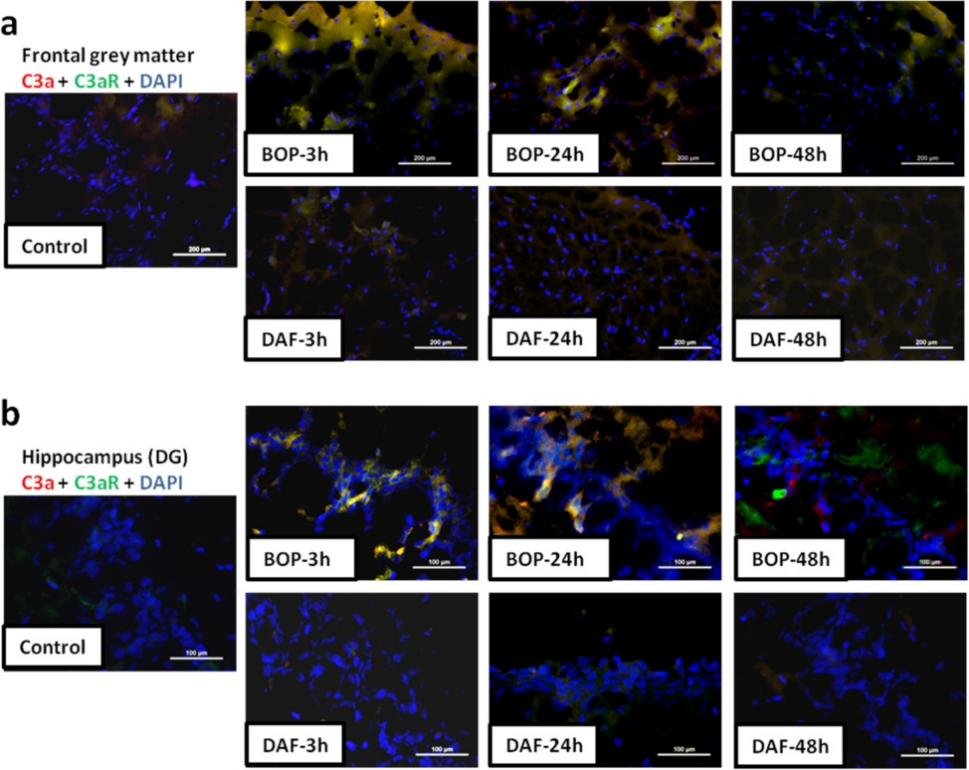
**DAF treatment reduces interaction of C3a-C3aR in the rat brain tissue after blast exposure.** Representative photomicrographs of C3a-C3aR interaction in frontal grey matter **(a)** and hippocampus (DG) **(b)** of frozen sections stained with anti-C3a (red) and anti-C3aR (green) antibodies. Original magnification of × 200 (grey matter) and × 400 (DG). Scale bars, 200 μm (grey matter) and 100 μm (DG). n = 8 for control, 3 and 24 h experimental groups. n = 5 for 48 h experimental groups.

Increased expression and interaction of C3a and C3a receptor (C3aR) were observed in both the cortex and hippocampus at 3 and 24 h after BOP when compared to respective controls (Figure 6a and b). In contrast, early injection of rhDAF at 30 min post BOP led to a significant reduction in the expression and interaction of C3a and C3aR in brain tissue at 3 h and 24 h after blast injury (Figure 6a and b).

### rhDAF inhibits systemic and local cytokine release in rats exposed to BOP

**Figure 7:**
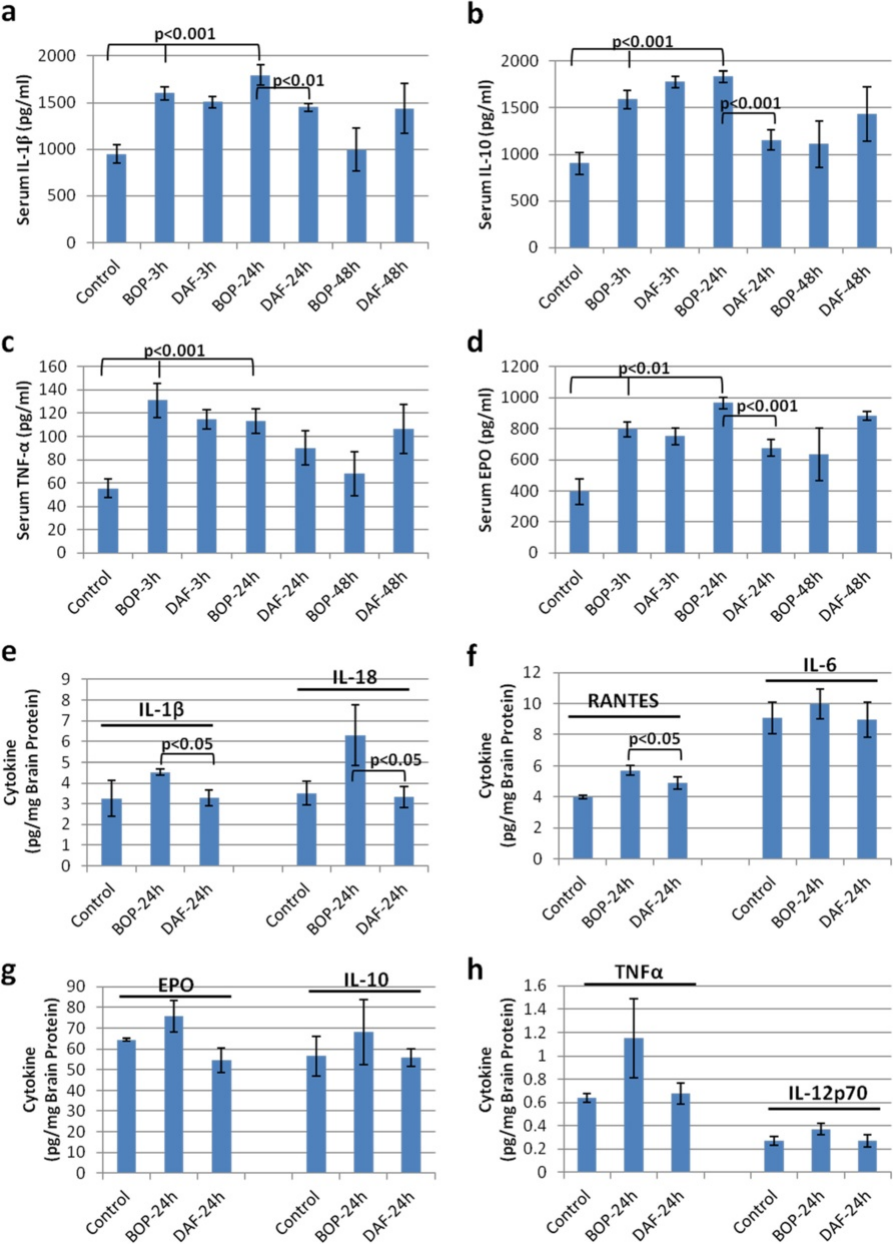
**DAF treatment decreases systemic and local cytokine levels in animals exposed to BOP.** Cytokine levels of blood serum **(a-d)** and brain frontal cortex **(e-h)** were measured by Luminex 200 using Bio-Plex Pro™ rat cytokine multiplex assay. Group data is expressed as mean ± SEM and compared using unpaired t-test. n = 8 for serum samples and n = 3 for brain tissues.

It has been well established that inflammation represents a common pathological reaction to TBI. In particular, production of multiple inflammatory cytokines and chemokines is one of the characteristics of TBI [3]. These changes occurred in the current model of moderate BOP in rats, where systemic pro- and anti-inflammatory cytokines were found to be released at significant levels as early as 3 h, reaching to peak at 24 h, and diminished at 48 h after BOP exposure. In particular, BOP triggered the release IL-1β, EPO, TNFα and IL-10 in the serum (Figure 7a-d). Interestingly, rhDAF treatment not only inhibited pro-inflammatory cytokine release (IL-1β and EPO) but also reduced anti-inflammatory cytokine (IL-10) production after the blast exposure. The release of these cytokines were slightly reduced at 3 h, and significantly attenuated at 24 h after BOP by an early bolus administration of rhDAF (Figure 7a-d).

Similar findings were found in local production of cytokines in the rat cortex. Specifically, exposure to BOP significantly elevated levels of RANTES at 24 h after blast (Figure 7f). Although not significant, there was a trend towards increased levels of IL-1β, IL-18, IL-12p70, IL-6, IL-10, TNFa, and EPO, at 24 h after blast injury (Figure 7e-h). This observed increase of IL-1β, TNFα, RANTES, and IL-18 returned back to control levels after rhDAF treatment.

### rhDAF reduces phosphorylated tau in rat brain after blast exposure

**Figure 8:**
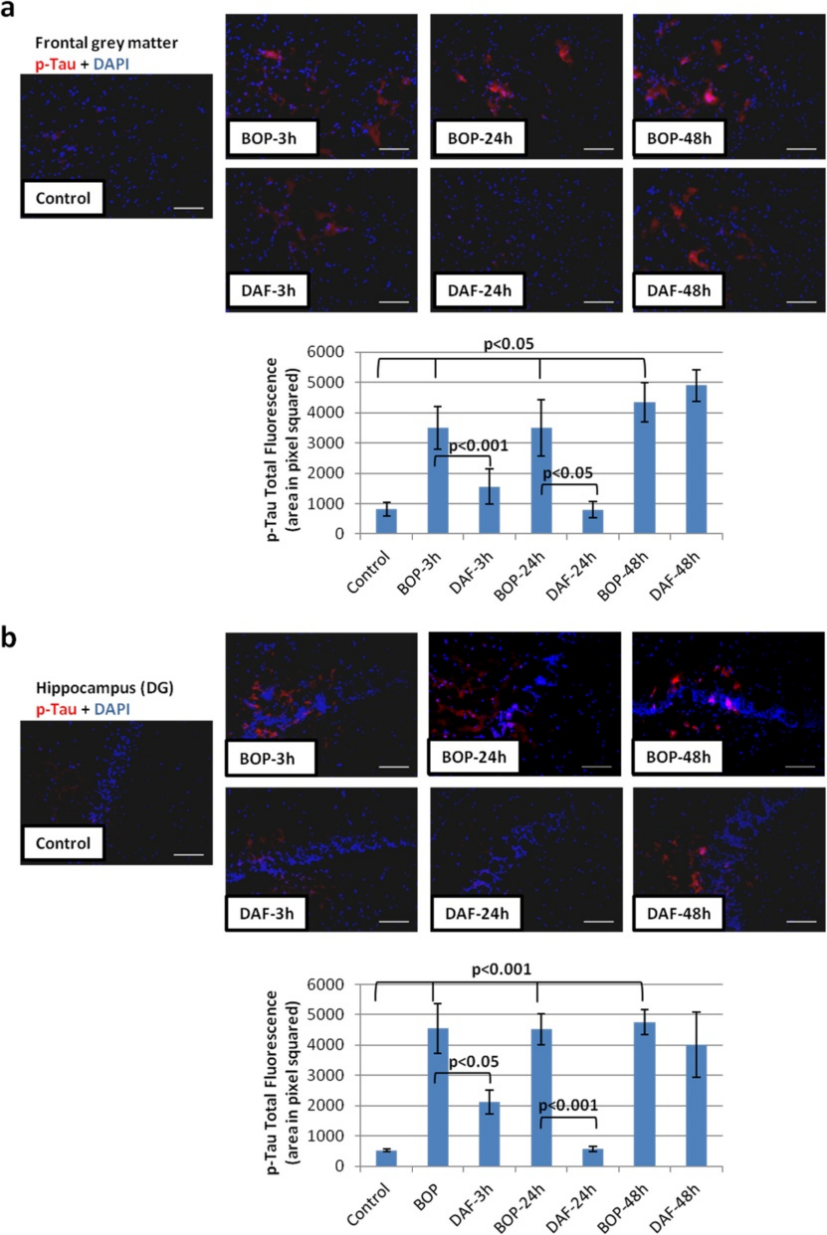
**DAF treatment decreases tau phosphorylation in animal brain tissue after BOP exposure.** Representative photomicrographs of frozen brain sections stained with anti-p-tau antibody (top) and the total fluorescence of p-tau (bottom). Frontal grey matter **(a)** and hippocampus (DG) **(b)**. Original magnification of × 200. Scale bar, 200 μm. Group data is expressed as mean ± SEM and compared using one-way ANOVA followed by Bonferroni test. n = 8 for control, 3 and 24 h experimental groups. n = 5 for 48 h experimental groups.

It has been well established that hyperphosphorylation of tau (p-tau) is involved in various neurodegenerative diseases [24]. A recent report presented evidence of accumulation of p-tau in mice blast TBI [25]. We investigated the effects of blast on tau phosphorylation in the cortex and in the dentate gyrus area of the hippocampus in our blast model. Phosphorylated tau immunoreactivity was observed in the cortex and hippocampus as early as 3 h and increased up to 48 h after blast in comparison to the controls (Figure 8a and b). Interestingly, administration of rhDAF attenuated the increased phosphorylation of tau at 3 and 24 h post blast (Figure 8a and b). However, rhDAF administration had little effect on the expression and accumulation of p-tau at 48 h after blast exposure.

### rhDAF decreases aquaporin-4 (AQP-4) expression in cerebral cortex

**Figure 9:**
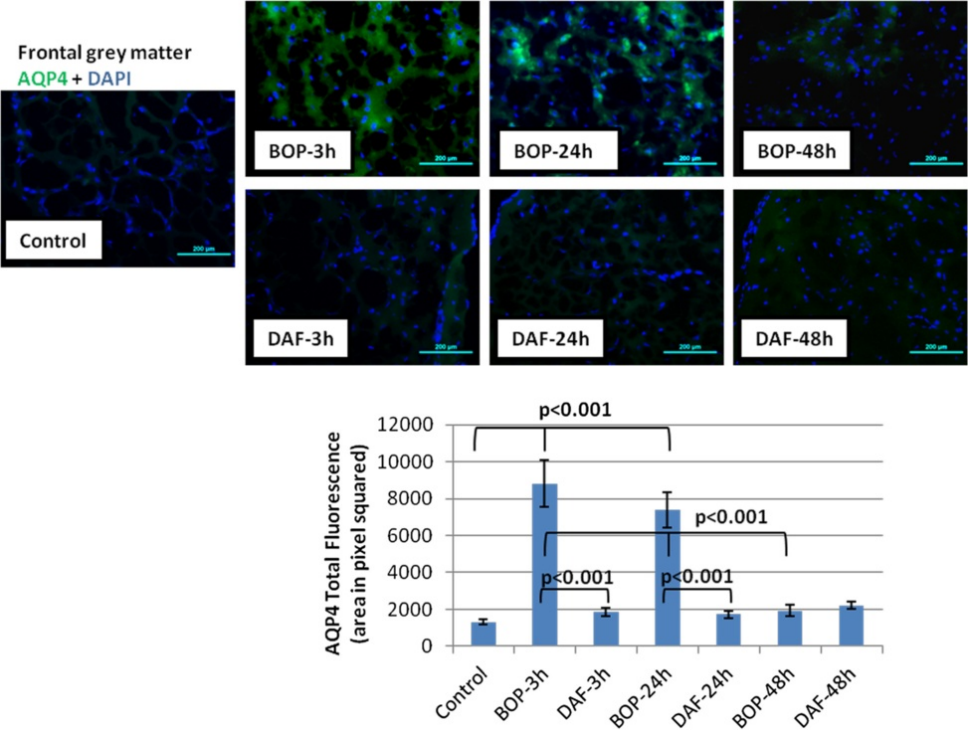
**DAF treatment reduces expression of aquaporin-4 in the animal brain cortex post BOP exposure.** Representative photomicrographs of frozen brain sections were immunostained with anti-aquaporin-4 antibody (top panel) and the total fluorescence of aquaporin-4 (bottom panel) Original magnification of × 200. Scale bar, 200 μm. Group data is expressed as mean ± SEM and compared using one-way ANOVA followed by Bonferroni test. n = 8 for control, 3 and 24 h experimental groups. n = 5 for 48 h experimental groups.

Increased expression and upregulation of AQP-4 has been associated with brain cytotoxic edema in TBI models [26]. Consist with our previous findings [22], BOP led to significant increases in AQP-4 expression in the cortex at 3 and 24 h after injury (Figure 9). Noteworthy, treatment with rhDAF significantly reduced AQP-4 expression compared to the untreated group after blast exposure (Figure 9).

### rhDAF protects the blast-induced BBB vascular permeability

**Figure 10:**
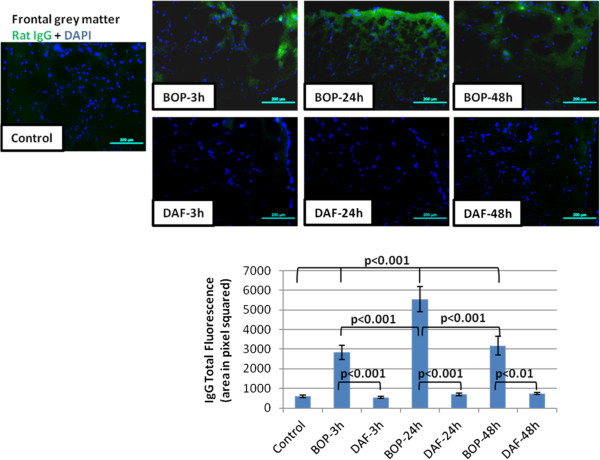
**DAF treatment decreases IgG immunoreactivity in brain cortical sections after BOP exposure.** Representative photomicrographs of frozen sections stained anti-rat IgG antibody (top panel) and the total fluorescence of IgG (bottom panel). Original magnification of × 200. Scale bar, 200 μm. Group data is expressed as mean ± SEM and compared using one-way ANOVA followed by Bonferroni test. n = 8 for control, 3 and 24 h experimental groups. n = 5 for 48 h experimental groups.

Traumatic brain injury disrupts the BBB integrity leading to extravasation of inflammatory proteins and infiltration of immune cells into the brain, and subsequent neuroinflammation and neurodegeneration. The spatial extravasation of endogenous IgG immunoreactivity was used as an index for BBB breakdown following BOP exposure [27]. Qualitative assessment of IgG extravasation revealed a significant increase in IgG immunoreactivity in the outer most layer of the cortex at 3, 24, and 48 h after BOP exposure (Figure 10). Treatment with rhDAF significantly reduced the extravasation of IgG at 3, 24 and 48 h after blast exposure (Figure 10), indicating its potential in protection of BBB injury.

## Discussion

Previously, we have demonstrated that early complement activation and inflammatory response were associated with blast-induced neurotrauma in rats after a moderate BOP exposure (120 kPa) [22]. In this study, we evaluated the effects of complement inhibition on neuroprotection after blast injury. We found that: 1) administration of rhDAF 30 min after moderate blast exposure displays a protective effect on brain histological damage as well as BBB breakdown and brain edema during the first 24 and 48 h after BOP; 2) rhDAF treatment attenuates blast-triggered systemic complement activation at 3 h and local complement deposition during the first 48 h, as well as systemic and local cytokine release at 24 h after the injury; and 3) Treatment with rhDAF reduces blast-instigated C3a-C3aR interaction and tau phosphorylation during the first 24 h after the blast exposure. Taken together, the data presented here provides first line evidence of early complement inhibition as an effective therapeutic strategy for blast-induced neurotrauma and neuroinflammation.

DAF, a ubiquitously expressed intrinsic complement regulatory protein, inhibits complement activation by preventing the assembly or accelerating the disassembly of the C3/C5 convertases in both the classic and alternative pathways thereby limiting the local C3a/C5a and C5b-9 production [28]. Human DAF has a structure similar to rat DAF and has displayed cross-species reactivity [29]. The selected dosage of rhDAF was in the titrated range used in the previous studies of hypoxia in rat primary neuronal cells [12], mouse ischemia-reperfusion [23,28], swine hemorrhagic shock [30,31] and rat hemorrhagic shock (unpublished data). The time window for rhDAF administration (30 min) after blast injury used in this study was based on previous findings that systemic complement activation after a moderate BOP exposure paralleled BBB breakdown as early as 3 h, persisting up to 48 h, and returning to control levels by 72 h after the injury [12,22]. Intravenous administration of rhDAF accumulated in the brain cortex and hippocampus as early as 3 h and persisted up to 48 h following BOP (Figure 1). The distribution of rhDAF in the brain was associated with the cerebral endothelium, suggesting that it might bind to the damaged endothelium and enter into the brain through the breached BBB after the blast injury.

C3a-C3aR interaction is a common initiating signal for subsequent reactions that initiates an inflammatory response. C3aR, a G-protein coupled receptor, is constitutively expressed in the central nervous system, on both neurons and glia [32]. Significant up-regulation of C3aR in murine brain after ischemia has been observed [12,33]. Interaction of C3a-C3aR leads to enhanced and maintained inflammatory responses such as leukocyte infiltration, vascular permeability, leukocyte activation, and inflammatory cytokine production [34]. Consistent with our previous findings [12], increased C3aR expression and interaction of C3a-C3aR were observed in rats exposed to BOP. Notably, C3a-C3aR engagement was markedly reduced in rhDAF-treated BOP injured animals, presumably through limiting local expression of C3aR and C3a and/or preventing the extravasation of systemic C3a through increased integrity of the BBB.

TBI results in brain microvasculature and BBB damage, leading to increasing BBB permeability. BBB integrity was assessed by immunoglobulin (IgG) staining. Staining for immunoglobulin (IgG) is an effective technique for assessing BBB integrity as the breach of the BBB to plasma protein, such as IgG, is a prominent feature of experimental brain injury [35]. The increased BBB permeability observed during the initial 48 h after blast (Figure 10) is congruent with previously reported increases in IgG staining at 3 and 24 h following blast [27]. In contrast, in rhDAF treated animals a significant reduction in BBB permeability was observed. BBB limited permeability with rhDAF treatment could be one way in which local cytokines and complement activity is down-regulated.

Our previous and current data demonstrated increased deposition of C3 and C5b-9 associated with cortical vasculatures at 3 and 48 h after BOP exposure, suggesting complement deposition could be playing pivotal role in BBB disruption post-injury. In agreement, early rhDAF treatment reduced local complement deposition (C3 and C5b-9) 3, 24 and 48 h post-injury and concurrently reduced BBB permeability (Figures 5, 6 and 10). The paralleled change between BBB dysfunction and complement deposition (C3 and C5b-9) indicates that systemic complement activation may contribute to complement protein accumulation in the brain cortex via crossing the damaged BBB following blast injury. Nevertheless, BOP exposure might have also resulted in elevation of C3 transcription in the cortex, suggesting the late complement protein accumulation could be, at least partially, produced locally through a C3a-C3aR interaction.

Interestingly, treatment with rhDAF decreased systemic and local cytokine levels at 24 h but not at 3 h after blast. The delayed effect of rhDAF on systemic cytokine release suggests an indirect effect of DAF on cytokine production presumably through the interruption of the C3a-C3aR and/or C5a-C5a receptor (C5aR) interaction and subsequent attenuation of cytokine synthesis via down-regulation of inflammatory gene transcriptional activity [36-38]. However, the effect of rhDAF on local cytokine attenuation in the cortex could be due to both the inhibition of cytokine synthesis via the interruption of C3a-C3aR engagement and the decrease of extravasation of systemic cytokines through the enhancement of BBB integrity.

AQP4, the principal member of the aquaporin protein family in the central nervous system, is expressed in astrocyte processes of the glia limitants externa and in perivascular astrocyte foot processes. AQP4 plays a key role in cerebral cytotoxic edema formation [26]. We observed an increased expression of AQP4 in the brain during the first 24 h which correlated with early cytotoxic edema. AQP4 expression largely normalized 48 h after the blast injury, indicating that cytotoxic edema is an early event in blast-induced brain injury. It is interesting to note, complement system activation has been reported to enhance AQP4 expression [22] and the observed increase in complement activation after blast exposure may play an important role in brain cytotoxic edema induced by AQP4. In agreement, our data shows that rhDAF exerted a protective effect on cytotoxic edema, and normalized blast-induced overexpression of AQP4. In addition, pro-inflammatory cytokines have been reported to induce the expression of AQP4 in rat astrocytes [39,40]. This regulatory effect of DAF on AQP4 expression could be at least partially explained by its role in attenuating the expression of IL-1β and IL-18, which has been shown to down-regulate AQP4 expression after TBI.

Increasing evidence suggests that TBI is associated with tauopathy, characterized by neurofibrillary tangles and neuropil threads composed of hyperphosphorylated tau [41]. Recent studies in mice with genetic deficiency of CD59 and Cr-related protein Y have demonstrated that the complement system has an active role in the development of tau pathology and neurodegeneration [42,43]. In the present study, hyperphosphorylated tau developed during the first 48 h after blast injury. These results are consistent with other reports which showed tauopathy in military personnel exposed to explosive blast as well as in blast-exposed mice [25]. Intriguingly, rhDAF treatment reduced early tau phosphorylation at 3 and 24 h, but not at 48 h after the blast. One possible explanation could be early tau phosphorylation, but not late tau phosphorylation, is complement activation-dependent. Another possibility is rhDAF, although present in the brain tissues 48 h after administration may not be functional to down regulate tau phosphorylation. Further studies will be needed to confirm these speculations.

Increased expression of C5aR was reported to be associated with enhanced phosphorylated tau in the brain of Alzheimer’s patients [44]. Moreover, treatment with a C5aR antagonist reduced the amyloid deposits and tau phosphorylation with enhanced neuronal functions in a murine model of Alzheimer’s disease [45]. Although increased expression of C5aR and interaction of C5a-C5aR was not observed in this study (data not shown), it is very likely that early tau phosphorylation is regulated by the enhanced C3a-C3aR signaling axis in our blast TBI model. Tau is also reported as a potent, antibody-independent activator of the classical complement pathway in chronic neurodegenerative disease [46,47]. Thus hyperphosphorylated tau, in turn, might lead to and/or maintain local cerebral complement activation after blast injury. Blast exposure has been considered to increase the risk for late development of chronic traumatic encephalopathy (CTE), a tau protein-linked neurodegenerative disorder, which is associated with post-traumatic stress disorder (PTSD) [20]. Consequently, the ability of tau to activate classical complement pathway could provide a mechanism for initiating and sustaining a chronic, low-level cerebral inflammatory response that may cumulate over the disease course and contribute to PTSD after blast injury. Future studies will be necessary to look into the relationship between complement and tauopathy at subacute and chronic phases of blast injury, and to determine whether complement inhibition has beneficial effects on blast-induced CTE.

## Conclusions

DAF protects against BINT by suppressing the systemic and local inflammatory response, reducing tau phosphorylation, improving BBB integrity, and decreasing cytotoxic edema. Modulation of the complement system after TBI is a novel and promising therapeutic tool aimed at manipulating secondary injury processes after BINT.

## Competing interests

The authors declare that they have no competing interests.

## Authors’ contributions

YL participated in the experimental design, performed the blast experiment, sampling, immunofluorescent staining, histological evaluation, data analysis, manuscript writing and formatting. MC participated in the animal experimental design and manuscript revision. JS performed the tissue cytokine assay, edited and formatted the manuscript. BL performed immunostaining, Fluoro-Jade B staining, CH50 assay, and blood and tissue cytokine assays. RMM and JDR provided critically important intellectual revision and scientific consultation. JJDL conceived the study, design and coordinated execution, performed data analysis and interpretation, wrote and gave final approval for manuscript submission. All authors read and approved the final manuscript.

## Disclaimer

Research was approved by the Institutional Animal Care and Use Committee and was conducted in compliance with the Animal Welfare Act as well as other federal statutes and regulations relating to animals and experiments involving animals and adheres to principles stated in the Guide for the Care and Use of Laboratory Animals, NRC Publication, 1996 edition.

The opinions or assertions contained herein are the private views of the authors and are not to be construed as official or reflecting the views of the US Department of the Army or The US Department of Defense.
